# Effect of suicidality on clinical and behavioural outcomes in HIV positive adults in Uganda

**DOI:** 10.1371/journal.pone.0254830

**Published:** 2021-08-20

**Authors:** Godfrey Zari Rukundo, Jonathan Levin, Richard Stephen Mpango, Vikram Patel, Eugene Kinyanda

**Affiliations:** 1 Department of Psychiatry, Mbarara University of Science and Technology, Mbarara, Uganda; 2 School of Public Health, Faculty of Health Sciences, University of the Witwatersrand, Johannesburg, South Africa; 3 Mental Health Project, MRC/UVRI and LSHTM Uganda Research Unit, Entebbe, Uganda; 4 Department of Mental Health, School of Health Sciences, Soroti University, Soroti, Uganda; 5 Department of Global Health and Social Medicine, Harvard Medical School, Boston, Massachusetts, United States of America; 6 Department of Psychiatry, Makerere College of Health Sciences, Kampala, Uganda; University of Washington, UNITED STATES

## Abstract

**Introduction:**

Suicidality is a risk of a person committing suicide often characterized by suicidal ideation, intent or attempts. Despite the high burden of suicidality among individuals living with HIV and HAIDS, there is paucity of data on the impact of suicidality on clinical (such as CD4 counts and HIV disease progression) and behavioural outcomes (such as adherence to HIV Medications). Cross-sectional investigations of these associations are often complicated by bidirectional causal relationships and hence the need for longitudinal study designs. We conducted a cohort study to determine the impact of suicidality on clinical and behavioural outcomes among adults living with HIV/AIDS in Uganda.

**Materials and methods:**

We conducted the study among 1099 ART naïve adults living with HIV/AIDS in Uganda. Data were collected at three time points: baseline, 6 and 12 months. Multiple regression and discrete time survival models were used to determine the relationship between suicidality and indices of HIV outcomes.

**Results:**

Majority of the participants were female and the participant mean age was 35 years. Most of them (73%) had primary or no formal education. The proportion of participants with suicidality decreased from 2.9% at baseline to roughly 1% both at month 6 and month 12. Of the investigated clinical and behavioural outcomes, baseline suicidality only had a negative impact on missing a dose of ART where the odds of missing a dose of ART were 8.25 (95% CI 2.45–27.71, p>0.01) times higher for participants with suicidality compared to those without suicidality. The following outcomes were not significantly impacted by baseline suicidality: HIV clinical stage, CD4 count and risky sexual behaviour.

**Conclusions:**

The fact that baseline suicidality significantly negatively impacted ART adherence calls for the incorporation of psychosocial interventions to target indices of psychological distress such as suicidality to improve HIV related outcomes.

## Introduction

Suicidal ideation and attempts often referred to as suicidality are major risk factors for future completed suicide [1]. Apart from being a risk factor for suicide, suicidality also has negative impact on the quality of life lived by the affected individuals and their families [2]. Individuals with physical illnesses which are associated with shame, long suffering or pain, such as HIV/AIDS, have a higher risk of depression and suicidality [3–5]. In a Ugandan cohort of individuals with HIV/AIDS, depression negatively impacted the clinical and behavioural outcomes of HIV [6]. However, there is paucity of data on the impact of persistent suicidality (suicidal ideation and attempts) on clinical outcomes (such as CD4 counts and WHO clinical stage of HIV) and behavioural outcomes such as adherence to HIV medications and health seeking behaviour. Previous studies on suicidality in HIV/AIDS, have generally considered HIV related clinical factors as predictors for suicide [7, 8]. However, the relationship could be bidirectional, with suicide worsening the clinical and behavioural outcomes in individuals living with HIV.

Cross-sectional investigations of these associations are often complicated by the bidirectional causal relationships and hence the need for longitudinal study designs. We conducted a cohort study to determine the impact of suicidality on clinical and behavioural outcomes among adults living with HIV/AIDS in Uganda. The findings of this study will help in the identification of possible intervention strategies such as integration of suicidality assessment and management into routine HIV care.

## Materials and methods

### Study design and site

This was a prospective cohort study among adults living with HIV (PLWH), attending two specialized HIV clinics, the semi-urban site clinic in Entebbe’ and the ‘predominantly rural clinic in Masaka, run by The AIDS Support Organisation (TASO) in Uganda [9].’ The study was conducted from May 2012 to December 2013. Data collection was undertaken at three time points: baseline (when participants undertook their first study interview after enrolment into the study) 6-months after the baseline assessment and 12-months after baseline assessment. Initiation of ART was implemented by TASO independently of the study. At the time of the study, national treatment guidelines for HIV-infected individuals recommended the initiation of ART at a CD4 cell count of below 250 cells / μl. In addition, individuals initiating ART were required to have identified an appropriate treatment supporter.

### Sampling procedure

**Fig 1:**
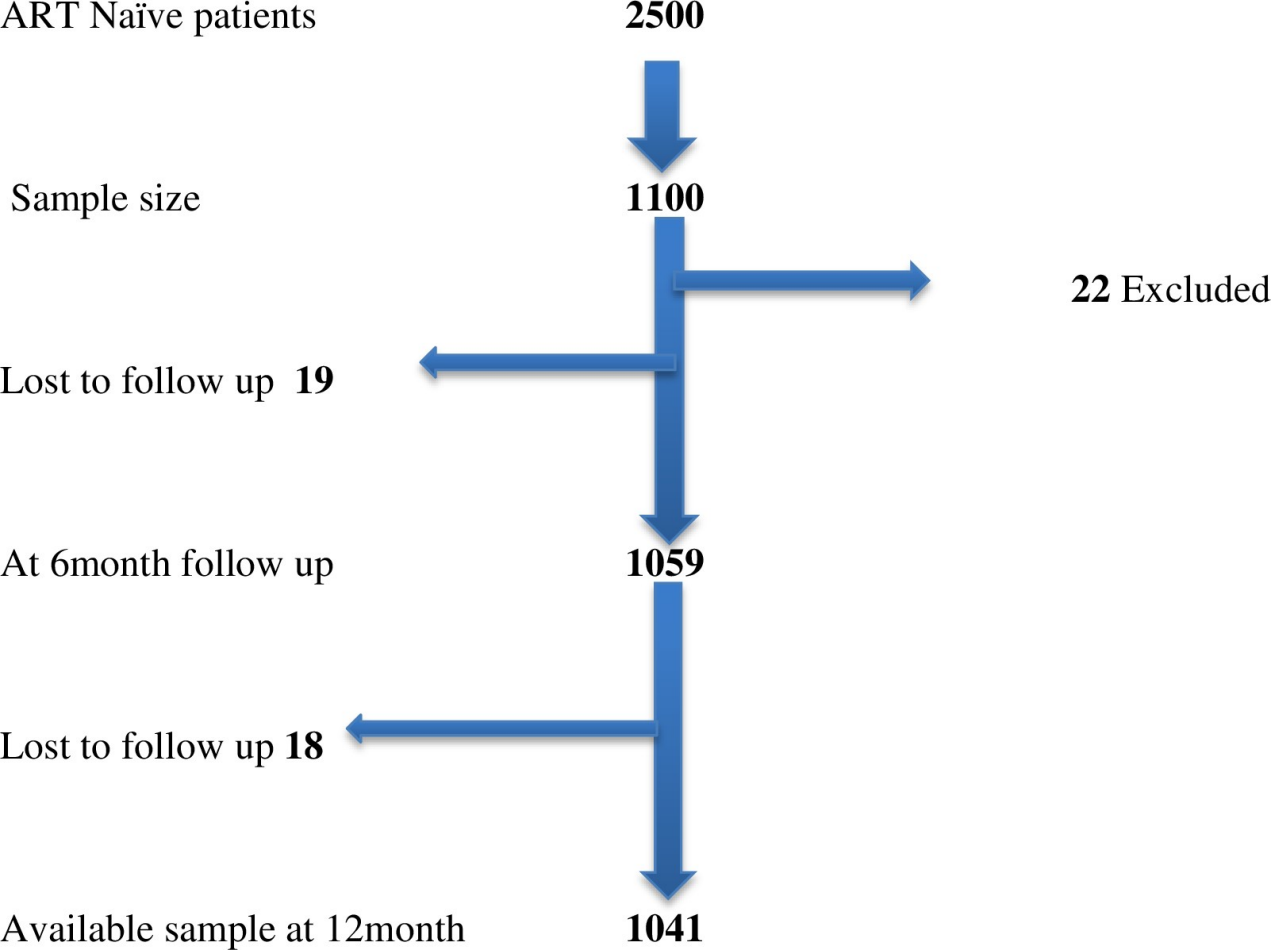
Flow chart showing participant recruitment data collection tools. This figure shows the initial sample size of 1100 and the participants who were excluded and those who were lost to follow up at 6 months and at 12 months. Entries for one participant were found to be incomplete and were not included in the final analysis which was done for 1099 instead of 1100 participants.

At the time of the study, TASO clinic in Entebbe had 7000 active clients of whom about 3000 were ART naïve while TASO clinic in Masaka had 6,000 active clients of whom about 2,500 were not on ART. This study aimed to enroll 1100 ART naïve HIV-infected adults from the two clinics. The sample was selected by simple random sampling; 1100 at baseline, 1059 at six months follow up and 1041 at 12 months follow up (Fig 1). In order to determine the effect of suicidality on clinical and behavioural outcomes, we aimed to detect an effect size of 0.15 with the power of 90%, α of 0.05 (two sided), β of 0.1 [6]. To obtain the required sample from the two HIV clinics, a sub-register of all active but HIV naïve clients was created. From these sub-registers a random sample of ART naïve patients was recruited from each study clinic using a table of random numbers until a combined total study sample of 1100 was obtained.

Risky sexual behavior was measured by at least one affirmative answer to the 5 questions on sexual behaviour. The questions included: ‘in the last month, have you had sex with anyone other than your regular partner?’ Among those who practiced high risk sexual behaviours ‘did you always use condoms?’ ii) have you had sex in exchange for gifts/money? iii) Have you had forced sex including rape? iv) Have you had sex with someone much older/younger than you? v) Have you had sex with someone you had just met?)’.

The inclusion criteria for this study were: i) a person living with HIV/AIDS who was ART naïve and registered with the outpatient clinic at either TASO Entebbe and TASO Masaka clinics; ii) aged at least 18 years old at enrolment; iii) conversant in Luganda, the language in which the study instruments were translated. Exclusion criteria were patients who were too sick or unable to understand the study instruments, and those who had missed their most recent scheduled clinic visit. Eligible participants (Fig 1) were recruited after they had provided written informed consent after explanation of the study objectives and procedures. About 2% of the selected patients could not be recruited into this study because of any one of the following reasons: i) were already enrolled in another study or ii) refused to participate in the study for any other reason.

Individuals found to have significant psychiatric problems requiring specialist assessment and management were referred to psychiatric departments/units nearest to the study sites.

The data collection tools consisted of structured and standardised locally translated psychosocial assessment instruments, most of which have previously been used among persons living with HIV (PLWH) in Uganda by this study group [10]. The study variables were: socio-demographic factors: these included study site, sex, age, highest educational attainment, marital status, religion, occupation and socio-economic index (SES index) [10]. 2) Psychosocial factors (coping style, negative life events and social support) were some of the variables in this study. We considered mean scores for each of the three variables 3) Exposure variable (suicidality) defined as having serious suicidal ideation and or suicidal attempt in the past twelve months from the date of assessment as determined by the section on suicide in the Diagnostic Statistical Manual IV–based suicidality module of the Mini International Neuropsychiatric Interview (M.I.N.I) [11]. Suicidality assessments were considered for each of the 3 time points and were reported as a binary outcome with respondents reporting as either having suicidality or not having suicidality. A diagnosis of suicidality was made if the respondent answered yes to the question on suicidal ideation or the one on suicidal attempt or both questions. 4) Indices of HIV related outcome measures: i) HIV disease progression [CD4 counts, WHO Clinical Staging criteria) [16]; ii) health seeking behaviour (number of visits to health facilities in last month; iii) adherence to HIV medications [three day antiretroviral therapy pill count recall [12, 13]; and iv) risky sexual behaviour [6].

### Statistical analysis

The impact of suicidality on the four HIV-related outcome domains (HIV disease progression, adherence to HIV medications, health seeking behaviour and risky sexual behaviour) was investigated using five outcome variables: i) CD4 count at visit 2 ii) Having experienced a WHO stage 3 or 4 event at month 6 or month 12 iii) Having missed at least one dose of ART medications in the three days prior to the interview iv) The time to the first visit to a health facility-measure of health seeking behaviour and v) Having engaged in risky sexual behaviour. Risky sexual behavior was measured by at least one affirmative answer to the 5 questions on sexual behaviour. The questions included: ‘in the last month, have you had sex with anyone other than your regular partner?’ Among those who practiced high risk sexual behaviours ‘did you always use condoms?’ ii) have you had sex in exchange for gifts/money? iii) have you had forced sex including rape?; iv) have you had sex with someone much older/younger than you?; v) have you had sex with someone you had just met?)’. Entries for one participant were found to be incomplete and were not included in the final analysis which was done for 1099 instead of 1100 participants that were initially enrolled.

***i) CD4 count at visit 2 (6 months) and visit 3 (12 months)*.** Suicidality at baseline was used as the exposure variable at month 6, whereas suicidality at month 6 was used as the exposure variable at month 12. Multiple linear regression models were fitted with the use of robust standard errors to account for the correlation between CD4 counts within participants. The analysis adjusted for study site, sex, age at baseline, visit (i.e. month 6 or month 12), and baseline CD4 cell count as explanatory variables. Participants who initiated ART between baseline and month 6 were excluded, whereas participants who initiated ART between month 6 and month 12 were included at month 6 but excluded at month 12. The measure of association for CD4 count is the difference in CD4 cell count (cells/mL) between participants with suicidality (at the previous visit) and those without suicidality.

***ii) Having experienced a WHO stage 3 or 4 event at month 6 or month 12*** was also used to measure the domain HIV disease progression. The time to the first WHO stage 3 or 4 event was analyzed using discrete time survival models [14]. Discrete time survival models were used to investigate the time (6 months or 12 months) to the first WHO stage 3 or 4 event. The primary exposure, suicidality, was lagged, that is, suicidality at baseline was used as the exposure variable for a WHO stage 3 or 4 event at month 6 (characterized by weight loss of more than 10%, chronic diarrhea for more than a month, fevers for more than a month, oral thrush, oral hairy leucoplekia, TB within the last 1 year, and severe bacterial infections like pneumonia and pyomyositis). The analysis adjusted for study site, sex, age, visit (ie, month 6 or month 12), initiation of ART and baseline CD4 cell count as explanatory variables. Participants who initiated ART before month 6 were excluded, whereas those who initiated ART between month 6 and month 12 were included at month 6 but excluded at month 12. Participants who had already experienced a WHO stage 3 or 4 event at baseline were also excluded from the analysis. The measure of association is the (adjusted) odds ratio for a WHO stage 3 or 4 event for participants with suicidality (at the previous visit) compared with those without suicidality.

***iii) Having missed at least one dose of ART medications*** in the 3 days before the interview was used as a measure for the domain on adherence to HIV medication. Missing at least one dose of ART at month 6 and month 12 was analyzed by fitting a multiple logistic regression model to a “long” data set with up to 2 observations per participant; robust standard errors were used to account for the correlation of responses within participants. In this case, the primary exposure (suicidality) was not lagged, since the suicidality was evaluated over the 2 weeks before the visit and missing at least one dose of ART was evaluated over the 3 days before the visit, so we assumed that the exposure (suicidality) preceded the outcome (missing at least one dose of ART). The analysis adjusted for study site, sex, age, visit (i.e. month 6 or month 12), and baseline CD4 cell count as explanatory variables. The analysis was restricted to participants who initiated ART between baseline and month 6 (who were included at month 6 and month 12) and participants who initiated ART between month 6 and month 12 (who were included at month 12 only). The measure of association is the (adjusted) odds ratio for missing at least one dose of ART for participants with suicidality compared with those without suicidality.

(***iv)*. *The time to the first visit to a health facility*** was used as a measure of health-seeking behavior and was analyzed using odds of occurrence [15]. The primary exposure, suicidality, was lagged, that is, suicidality at baseline was used as the exposure variable for a visit to a health facility at month 6, whereas suicidality at month 6 was used as the exposure variable for a visit to a health facility at month 12. The analysis adjusted for study site, sex, age, visit (i.e., month 6 or month 12), and baseline CD4 cell count as explanatory variables. Participants who had their first visit to a health facility at baseline were excluded from the analysis, whereas those who visited a health facility between month 6 and month 12 were included at month 6 but excluded at month 12. The measure of association is the (adjusted) odds ratio for a visit to a health facility for participants with suicidality (at the previous visit) compared with those without suicidality.

***v) Having engaged in risky sexual behavior*** (as measured by at least one affirmative answer to the 5 questions on sexual behavior) was analyzed using discrete time survival models. The primary exposure (suicidality) was lagged. The analysis adjusted for study site, sex, age, visit, and baseline CD4 count as explanatory variables. Participants who had engaged in risky sexual behavior at baseline were excluded from the analysis, whereas those who engaged in risky sexual behavior at month 6 were excluded at month 12. The measure of association is the (adjusted) odds ratio for risky sexual behavior for participants with suicidality (at the previous visit) compared with those without suicidality. We did not adjust for multiple significance testing. Although this increases the chance of type I errors, the aim of the analysis was to identify potentially detrimental consequences of suicidality; this can be seen as analogous to safety analysis in drug trials in which the aim is to identify potential risks caused by the investigational drug, in which case adjusting for multiplicity is not recommended [16, 17].

### Ethics approval and consent to participate

The study obtained ethical approval from the Uganda Virus Research Institute’s Science and Ethics Committee and the Uganda National Council of Science and Technology. Study participants were invited to consent and participate in this study by trained psychiatric nurses / psychiatric clinical officer(s) after being provided with adequate information about the study. Respondents found to have significant psychiatric problems were referred to psychiatric departments nearest to their study sites for further assessment and management.

## Results

At month 12 of follow up, the overall retention in the study was high 94.7% of the 1099 participants assessed at baseline. About 6% (n = 67) were lost to follow-up, of whom 1.6% (n = 18) were confirmed to have died during the course of this study. The CD4 cell count increased significantly with time, the amount of risky sexual activity reduced, number of health facility visits decreased and the rate of suicidality also significantly reduced at both 6 months and 12 months of follow up (Table 1). Majority of the participants were female and the participant mean age was 35 years. Most of them (73%) had primary or no formal education. A detailed description of the characteristics of this study population can be found in an earlier publication [6]. The number of participants at the two study sites was similar throughout the three reporting periods (baseline, 6 months and 12 months).

**Table 1:** Socio-demographics, suicidality and HIV related outcomes of the study population by data collection time period.

Factor	Level	Baseline	Month 6	Month 12
Overall		1099	1059	1041
**Socio-demographics**				
Study Site	Entebbe	542 (49.3%)	520 (49.1%)	509 (48.9%)
	Masaka	557 (50.7%)	539 (50.9%)	532 (51.1%)
Sex	Male	252 (22.9%)	243 (23.0%)	238 (22.9%)
	Female	847 (77.1%)	816 (77.0%)	803 (77.1%)
Age	Mean (s.d.)	35.1 (9.3)	35.1 (9.3)	35.1 (9.1)
	Median (IQR)	34 (28–41)	34 (28–41)	34 (28–41)
Age (grouped)	18–29	339 (30.8%)	321 (30.3%)	316 (30.4%)
	30–34	252 (22.9%)	248 (23.4%)	244 (23.4%)
	35–39	197 (17.9%)	188 (17.8%)	185 (17.8%)
	40–49	225 (20.5%)	218 (20.6%)	216 (20.8%)
	> = 50	86 (7.8%)	84 (7.9%)	80 (7.7%)
Education Status	None	120 (10.9%)	113 (10.7%)	113 (10.8%)
	Primary	680 (61.9%)	654 (61.8%)	641 (61.6%)
	Secondary or more	296 (26.9%)	289 (27.3%)	284 (27.3%)
	Missing	3 (0.3%)	3 (0.3%)	3 (0.3%)
Marital Status	Currently married	563 (51.2%)	540 (51.0%)	533 (51.2%)
	Widowed	163 (14.8%)	157 (14.8%)	158 (15.2%)
	Separated / divorced	267 (24.3%)	261 (24.6%)	254 (24.4%)
	Single	104 (9.5%)	99 (9.4%)	94 (9.0%)
	Missing	2 (0.2%)	2 (0.2%)	2 (0.2%)
Religion	Catholic	586 (53.3%)	566 (53.4%)	562 (54.0%)
	Protestant	237 (21.6%)	228 (21.5%)	224 (21.5%)
	Muslim	163 (14.8%)	158 (14.9%)	152 (14.6%)
	Seventh Day	16 (1.5%)	16 (1.5%)	14 (1.3%)
	Born Again	93 (8.5%)	87 (8.2%)	85 (8.2%)
	Other	4 (0.4%)	4 (0.4%)	4 (0.4%)
Occupation	Farmer / Fishing	324 (29.5%)	321 (30.3%)	310 (29.8%)
	Professional / clerical	43 (3.9%)	42 (4.0%)	42 (4.0%)
	Trader / Artisan / transport	396 (36.0%)	386 (36.4%)	383 (36.8%)
	Unemployed / Retired / housewife	139 (12.6%)	126 (11.9%)	126 (12.1%)
	Student / other	187 (17.0%)	174 (16.4%)	172 (16.5%)
	Missing	10 (0.9%)	10 (0.9%)	8 (0.8%)
SES index	Mean (s.d.)	15.1 (3.6)	15.1 (3.6)	15.1 (3.6)
	Median (IQR)	15 (13–17)	15 (13–17)	15 (13–17)
CD4 count	Mean (s.d.)	516.2 (267.6)	560.6 (235.4)	600.6 (233.4)
	Median (IQR)	471 (352; 665)	517 (407; 687)	556 (435; 711)
	Geometric mean (95% C.I.)	430.7 (412; 450)	514.6 (498; 532)	516.1 (497; 537)
WHO stage	I	533 (48.5%)	308 (45.4%)	233 (43.3%)
	II	500 (45.5%)	347 (51.2%)	265 (49.3%)
	III / IV	66 (6.0%)	23 (3.4%)	25 (4.6%)
	Missing	0	0	15 (2.8%)
Missed a dose of ART in past three days	No		289 (86.8%)	390 (83.3%)
	Yes		30 (9.0%)	31 (6.6%)
	N/A		14 (4.2%)	47 (10.0%)
Visits to health facility in past month	None	781 (71.1%)	603 (80.1%)	507 (86.7%)
	Once	146 (13.3%)	70 (9.3%)	47 (8.0%)
	Twice	85 (7.7%)	45 (6.0%)	18 (3.1%)
	Three or more	86 (7.8%)	31 (4.1%)	10 (1.7%)
	Missing	1 (0.1%)	4 (0.5%)	3 (0.5%)
Any Risky sexual activity	No	950 (86.4%)	832 (91.0%)	737 (90.9%)
	Yes	149 (13.6%)	74 (8.1%)	66 (8.1%)
	Missing	0	8 (0.9%)	8 (1.0%)

The proportion of participants with suicidality decreased from 2.9% (n = 32) at baseline to roughly 1% both at month 6 (n = 9, 0.9%) and month 12 (n = 12, 1.2%). The CD4 count increased over time–this increase was largely artefactual since those who started ART were excluded from subsequent analysis of CD4 count. Few participants experienced a new WHO stage 3 or 4 event, 6% (n = 66) at baseline, 3.4% (n = 23) at month 6 and 4.6% (n = 25) at month 12 (Table 2).

**Table 2:** Suicidality and HIV related outcomes of the study population by data collection time period.

Factor	Level	Baseline	Month 6	Month 12
Overall		1099	1059	1041
**Psychosocial exposures**				
Suicidality	No	1067 (97.1%)	1041 (98.3%)	1020 (98.0%)
	Yes	32 (2.9%)	9 (0.9%)	12 (1.2%)
	Missing	0	9 (0.9%)	9 (0.9%)
**HIV related outcomes**				
CD4 count	Mean (s.d.)	516.2 (267.6)	560.6 (235.4)	600.6 (233.4)
	Median (IQR)	471 (352; 665)	517 (407; 687)	556 (435; 711)
	Geometric mean	430.7	514.6	516.1
WHO stage	I	533 (48.5%)	308 (45.4%)	233 (43.3%)
	II	500 (45.5%)	347 (51.2%)	265 (49.3%)
	III / IV	66 (6.0%)	23 (3.4%)	25 (4.6%)
	Missing	0	0	15 (2.8%)
Patients who initiated ART				
Missed a dose of ART in past three days	No		289 (86.8%)	390 (83.3%)
	Yes		30 (9.0%)	31 (6.6%)
	N/A		14 (4.2%)	47 (10.0%)
Visits to health facility in past month	None	781 (71.1%)	603 (80.1%)	507 (86.7%)
	Once	146 (13.3%)	70 (9.3%)	47 (8.0%)
	Twice	85 (7.7%)	45 (6.0%)	18 (3.1%)
	Three or more	86 (7.8%)	31 (4.1%)	10 (1.7%)
	Missing	1 (0.1%)	4 (0.5%)	3 (0.5%)
Any Risky sexual activity	No	950 (86.4%)	832 (91.0%)	737 (90.9%)
	Yes	149 (13.6%)	74 (8.1%)	66 (8.1%)
	Missing	0	8 (0.9%)	8 (1.0%)

### Associations between psychosocial factors and HIV clinical and behavioural outcomes

The results of fitting models for the associations between psychosocial exposures and HIV related clinical and behavioural outcomes are shown in Tables 3 and 4. Table 3 summarizes the fitting of the multiple linear regression models to the log (CD4) counts at months 6 and 12. None of the associations between suicidality and log (CD4) count approached statistical significance. Of the investigated clinical and behavioural outcomes, suicidality only had a negative impact on missing a dose of ART where the odds of missing a dose of ART were 8.25 (95% CI 2.45–27.71, p>0.01) times higher for participants with suicidality compared to those without suicidality. The following outcomes were not significantly impacted by baseline suicidality: HIV clinical stage, CD4 count and risky sexual behaviour.

**Table 3:** Regression models for log (CD4) counts (effects expressed as geometric mean ratios).

Factor	Level	Effect as GMR (95% CI)*	P-value
Study Site	Entebbe	1 (baseline)	<0.00
	Masaka	1.11 (1.06; 1.16)	
Sex	Male	1 (baseline)	0.02
	Female	1.07 (1.01; 1.14)	
Age	Per 10 year increase	0.99 (0.96; 1.01)	0.33
Month	Per 6 month increase	1.06 (1.02; 1.09)	0.00
Baseline CD4 count	Per 1 log_10_ increase	2.63 (1.95; 3.54)	0.01
Suicidality	No	1 (baseline)	0.16
	Yes	1.12 (0.96; 1.32)	

**Note** * Effects shown in this column are adjusted for socio-demographic factors and baseline CD 4 counts.

**Table 4:** Logistic regression models for missed ART doses with robust estimation of variance.

Factor	Level	Odds Ratio (95% CI)*	P-value
Study Site	Entebbe	1 (baseline)	0.02
	Masaka	0.46 (0.25; 0.88)	
Sex	Male	1 (baseline)	0.43
	Female	0.75 (0.45; 1.26)	
Age	Per 10 year increase	0.97 (0.72; 1.31)	0.86
Month	Per 6 month increase	0.75 (0.45; 1.26)	0.28
Suicidality	No	1 (baseline)	0.00
	Yes	8.25 (2.45; 27.71)	

**Note 1** The social support variable used here is 2500/ (Social Support).

* Effects shown in this column are adjusted for socio-demographic factors and baseline CD4 counts.

* NB: IQR-Interquartile range, GMR- Geometric Mean Ratio, SD-standard deviation.

Table 4 summarizes the results of fitting logistic regression models for having missed a dose of ART in the three days before the visit. The odds of missing a dose of ART were 8.25 (95%CI 2.45–27.71, p>0.01) times higher for participants with suicidality compared to those without suicidality.

Table 5 summarizes the results of fitting discrete-time survival models for the outcomes of time to first new WHO stage 3/4 event, time to visit a health facility, and time to risky sexual behaviour.

**Table 5:** Discrete time survival models for various clinical and behavioural outcomes.

Factor	Level	Odds Ratio (95% CI)*	P-value
**New WHO stage 3 / 4 event**			
Negative coping style scores	Per 1 s.d. increase	1.40 (1.05; 1.88)	0.02
Social support scores	Per 1 s.d. increase	0.97 (0.72; 1.31)	0.83
Negative life events scores	Per 1 s.d. increase	0.80 (0.57; 1.10)	0.17
Suicidality	No	1 (baseline)	0.73
	Yes	1.44 (0.18; 11.29)	
**Time to visit a health facility**			
Negative coping style scores	Per 1 s.d. increase	0.89 (0.77; 1.03)	0.12
Social support scores^2^	Per 1 s.d. increase	1.16 (1.06; 1.27)	0.00
Negative life events scores	Per 1 unit increase	1.26 (1.09; 1.46)	0.00
Suicidality	No	1 (baseline)	0.07
	Yes	2.49 (0.93; 6.67)	
**Time to risky sexual behaviour**			
Negative coping style scores	Per 1 s.d. increase	1.12 (0.94; 1.33)	0.22
Social support scores	Per 1 s.d. increase	1.08 (0.89; 1.30)	0.44
Negative life events scores	Per 1 unit increase	0.99 (0.84; 1.19)	0.97
Suicidality	No	1 (baseline)	0.39
	Yes	0.42 (0.056; 3.13)	

**Note:** The social support variable used here is 2500/ (Social Support)

* Effects shown in this column are adjusted for socio-demographic factors and baseline CD4 counts only. IQR-Interquartile range, GMR- Geometric Mean Ratio, SD-standard deviation.

Following the findings by Ironson et al [18] in their study on psychosocial factors that predicted CD4 and viral load change, the results can be quantified by looking at the Odds ratios (OR) for an individual at the 75th percentile (P75) compared to a similar individual at the 25th percentile (P25) of the scale.

On time to the first new WHO stage 3 or 4 event, the significant exposure was negative coping style. Increasing risk for a new WHO stage 3 or 4 event was associated with increasing negative coping style scores (OR 1.61). On the results of fitting discrete-time survival models for the time to the first visit to a health facility, the significant psychosocial factors were social support, negative life events, and suicidality. The risk of visiting a health facility increased with: decreasing social support scores (nonlinear effect, OR for P25 vs. P75 was 1.12) and increasing negative life events scores (OR for P75 vs. P25 was 1.31).

The psychosocial factors that significantly impacted missing a dose of ART were social support, negative life events and suicidality. The odds of missing a dose of ART were higher for participants with: lower social support scores (OR for P25 vs. P75 was 1.21; the non-linear effect could be noted that this is similar to the OR for P10 vs. P25 which was 1.20 despite covering a much narrower range of participants) and higher negative life events scores (OR for P75 vs. P25 was 1.57).

## Discussion

This cohort study aimed to determine the effect of suicidality on clinical and behavioural outcomes among adults living with HIV/AIDS in Uganda. We found a positive association between suicidality and poor clinical and behavioural outcomes. Compared to previous studies, we looked at a broader range of psychosocial exposures and indices of HIV related outcomes. In this paper we discuss the effect of suicidality and psychosocial exposures on indices of HIV related outcomes with the latter grouped under the domains of HIV disease progression, drug adherence, health seeking behaviour and risky sexual behaviour.

In this study, suicidality predicted risky sexual behaviour. According to our knowledge, this is the first study that has investigated the impact of suicidality on clinical and behavioural outcomes among individuals with HIV in low resource settings. Previous studies have investigated the longitudinal impact of depression on HIV outcomes. For example, a study of depression by Kinyanda et al (2018) reported a negative association between depression and ART adherence and health seeking behaviour [6]. A study by Bhatia and colleagues in the united states of America also reported a negative relationship between depression and health seeking behaviour [19].

Two indices were used to assess HIV disease progression in this study, namely, CD4 count and time to the first new WHO stage 3 or 4 event. Only negative coping style was a significant predictor of time to the first WHO stage 3 or 4 event with none of the investigated exposures predicting trends in CD4 counts. Studies undertaken elsewhere have previously reported negative coping style as a predictor of faster HIV disease progression [20–22]. The relationship between psychosocial exposures and CD4 counts could have been confounded by extraneous factors given that CD4 counts in this study showed wide variability both between subjects and between time periods within subjects.

Health seeking behaviour in this study was assessed using time to first visit to a health facility. The psychosocial exposures which predicted increased utilisation of health facilities were poor social support, increasing number of negative life events and suicidality. The increased utilisation of HIV care services that was associated with the psychosocial exposures of poor social support, increasing negative life events and suicidality observed in this study may have been a ‘cry for help’ from persons experiencing psychosocial distress whose needs were not being met by an HIV care system that is not yet responsive to mental health and other psychosocial problems.

A limitation of this study is that some of the indices used to assess HIV related outcomes may have been confounded by factors outside the study. We attempted to control for this for one of the outcomes domain by having two indices to represent it. This however had to be weighed against the danger of having too many comparisons which would increase the risk of producing spurious results purely by chance. Therefore, to assess for the outcomes domain of HIV disease progression in this study, we used the indices of both CD4 cell counts (which showed extreme variability in this study) and the variable ‘new WHO stage 3 or 4 event’ (which showed more stability).

The investigated psychosocial exposures impacted all the investigated HIV related outcomes domains with suicidality impacting the most domains (three out of the four investigated HIV outcomes domains). These results suggest that to improve HIV related outcomes among patients accessing HIV care, there is a need to screen for and triage patients with significant mental health and psychosocial problems so that they can receive treatment for the mental health problems and additional psychosocial support to help them navigate through HIV care. Secondly, in order to optimise effectiveness of interventions aimed at improving HIV related outcomes, there may be a need to include components that address suicidality and other important psychosocial exposures such as the cognitive behavioural therapy for adherence and depression (CBT-AD) which has been shown to significantly improve depression and ART adherence outcomes [23]. However, since we know very little regarding the biological mechanisms underlying the observed relationships between exposures and outcomes [24] and that between suicidality and the other psychosocial exposures themselves, there is need for research in this area.

## Conclusions

The fact that baseline suicidality significantly and negatively impacted ART adherence calls for the incorporation of psychosocial interventions to target indices of psychological distress such as suicidality to improve HIV related clinical and behavioural outcomes.

## Supporting information

S1 Checklist (PDF)

S1 Data (DTA)
